# Relationship between red cell distribution width and prognosis of patients with osteosarcoma

**DOI:** 10.1042/BSR20192590

**Published:** 2019-12-20

**Authors:** Jian Zheng, Xiaopin Yuan, Weichun Guo

**Affiliations:** 1 Department of Orthopedics, RenMin Hospital of Wuhan University, Wuhan 430060, Hubei Province, China; 2 Cadre Health Service, RenMin Hospital of Wuhan University, Wuhan 430060, Hubei Province, China

**Keywords:** osteosarcoma, prognosis, red cell distribution width, Survival analysis

## Abstract

We retrospectively collected the clinical data and follow-up information of patients with osteosarcoma who were admitted to Department of Orthopedics, RenMin Hospital of Wuhan University from January 2010 to December 2016 and explore the relationship between red cell distribution width (RDW) and prognosis of patients with osteosarcoma. The present study finally included 271 patients with osteosarcoma with median follow-up time of 24.2 months (3–69 months). According to the RDW median, 135 patients belong to the low RDW group and 136 patients belong to high RDW group. Compared with low RDW group, the high RDW group tend to have metastasis (50 vs 32.6%, *P*=0.004), higher poor response rate to chemotherapy compared with the low RDW group (24.3 vs 7.4%, *P*=0.000) and higher C-reactive protein (CRP) (7.6 ± 4.9 vs 5.5 ± 4.5, t = 3.727, *P*=0.000). There was slightly significant difference in the types of pathology (χ^2^ = 8.059, *P*=0.045). The Kaplan–Meier analysis indicated survival curve of high RDW group was poorer than that in the low RDW group (*P*=0.020). The univariate cox analysis indicated that patients with RDW ≥ median had higher risk of poor prognosis compared with those who had RDW level < median (HR = 2.41, 95% confidence interval (CI): 1.51–3.83, *P*=0.000). After adjusting some potential cofounding factors, the elevated RDW was still associated with poor prognosis (HR = 1.66, 95% CI: 1.07–2.56, *P*=0.024). The elevated pretreatment RDW was associated with poor overall survival (OS) in patients with osteosarcoma and can be an independent predictor of prognosis.

## Introduction

Osteosarcoma is the most common primary malignant bone tumor so far. It originates from mesenchymal tissue and has a higher incidence and mortality than other primary bone tumors [1]. The osteosarcoma is one of the most common primary malignancies in children and adolescents, accounting for 2.4% of all pediatric malignancies. Approximately 20% of the patients had lung metastasis at the first visit, and approximately 40% had metastasis at the late stage of the disease [2,3]. The course of disease progressed rapidly, metastasis was early, recurrence rate was high, prognosis was very poor, 5-year survival rate was very low, and the 5-year survival rate of the patients with surgery alone was only 15–20% [4]. Over the past few decades, with the continuous improvement in limb salvage surgery and neoadjuvant chemotherapy and radiotherapy, the 5-year survival rate of patients with bone tumors has significantly increased by approximately 60–70% [5]. However, the long-term prognosis of patients with osteosarcoma is still poor. Approximately 80% of patients had recurrences after surgical treatment, and factors such as early lung metastasis and postoperative tumor recurrence were still bothering orthopedic clinicians [6]. Currently, the long-term survival rate of patients with recurrent osteosarcoma is less than 20%, although several molecular targeted drugs have emerged, none of them can be well used in the treatment of osteosarcoma, the emergence of chemotherapeutic drug resistance and side effects of chemotherapeutic drugs such as hepatoxicity, nephrotoxicity and cardiotoxicity [7]. These traditional therapies have reached a bottleneck in recent years. In addition, early diagnosis of bone tumors remains important. It is of great significance to seek a simple, convenient, sensitive and specific prognostic index for early detection of tumor and guidance of treatment decision.

Red cell distribution width (RDW) is a measurement of variability and size of erythrocytes, and is performed routinely as part of a complete blood cell count [8]. As an easy-to-measure inflammatory marker of systemic inflammatory response, RDW has been reported in many pathophysiological conditions including tumor. As we all know that inflammation in the tumor microenvironment is involved in the initiation, progression of cancer [9]. Recently, some studies also reported that RDW was associated with prognosis of several types of cancers. Yao et al. [10] reported that high pretreatment RDW levels in breast cancer patients were associated with poor overall survival (OS) and disease-free survival in a retrospective follow-up study with large sample size. In another study, Fukuokaya et al. [11] found that high RDW was significantly associated with presence of lymph node metastasis in patients with castration-resistant prostate cancer and was an independent predictor of worse treatment outcomes. This relationship was further confirmed in other cancers, including non-small cell lung cancer [12], esophageal cancer [13], laryngeal cancer [14] and ovarian cancer [15]. However, data about the role of RDW in the osteosarcoma are few, and previous studies give us some hints. In the present study, we explore the relationship between RDW and prognosis in the patients with osteosarcoma.

## Materials and methods

### Study population

We retrospectively collected the clinical data and follow-up information of patients who were admitted to Department of Orthopedics, RenMin Hospital of Wuhan University from January 2010 to December 2016. The patients met the following criteria: (1) patients received the standard chemotherapy plan: the first line (doxorubicin: 45 mg/m^2^dl-cisplatin:75–100 mg/m^2^d or 3 d) or MAP (high-dose methotrexate 8–12 g/m^2^+ doxorubicin: 45 mg/m^2^dl-2+ cisplatin:75–100 mg/m^2^d or 3 d) for at least six periods; (2) patients who received neoadjuvant chemotherapy, surgical excision and adjuvant chemotherapy (unless metastasis occurs during preoperative chemotherapy) were included; (3) all patients were diagnosed and confirmed via pathology examination. The following patients were excluded: patients were in stage IV and above. There was no definite diagnostic for tumor. No useful information exaction or follow-up results were obtained. Patients with systemic inflammatory, severe liver and kidney dysfunction, thromboembolic diseases, autoimmune diseases, hepatopathy, diabetes mellitus, hypertension, cardio- and cerebrovascular diseases, serious infection or other or other tumors were also excluded. The present study was approved by the Ethics Committee of RenMin Hospital of Wuhan University. The research was carried out in accordance with the World Medical Association Declaration of Helsinki, and all subjects provided written informed consent.

### Data collection

The clinical data were obtained from medical records. For each patient, the following data were collected: sex, age, body mass index, history of smoking, history of diseases, tumor size (≤5 vs >5 cm), clinical stage (I, II, III), the location of tumor (femur, tibia, humeral), metastasis or not, pathology types (osteoblastic, chondroblast, fibroblastic and telangiectatic) and response to chemotherapy (good vs poor). The clinical stage of osteosarcoma referred to the System for the Surgical Staging of Musculoskeletal Sarcoma. The diagnosis of osteosarcoma was confirmed by at least two physicians.

The blood routing examination was performed before patients received any treatments. This examination was competed via automatic biochemical analyzer. Hematologic testing was performed on the ADVIA 120 (Bayer Diagnostics, Newbury, Berkshire, U.K.) automated hematology analyzer, which measures hemoglobin photometrically, including white blood cell counts, platelet counts, hemoglobin and RDW, optical laser light scattering for cell enumeration, flow cytometer and laser diffraction for red blood cell counts. We also collected the triglycerides (TG), triglyceride, low-density lipoprotein cholesterol (LDL-C), high-density lipoprotein cholesterol (HDL-C), total cholesterol and C-reactive protein (CRP) levels.

### Follow-up and outcomes

In the present study, the enrolled patients were followed up by outpatient or telephone. The follow-up date was 31 December 2016. The median follow-up time was 24.2 months. Because this is a retrospective study, the accurate time of occurrence or metastatic was unavailable. We mainly treated the OS as the primary outcome, and the OS was defined as the interval from the date of diagnosis to date of death or last follow-up.

### Statistical analysis

We first divided the study population into two groups according to the median of RDW level (median = 13.6). Means and its standard deviation were used for continuous variables with normal distribution, and the independent *t* test was used for the comparison between cognitive impairment group and control group. For abnormal distribution, median with maximum and minimum was used for unnormal distribution and Wilcoxon rank sum test was used for comparison. For category data, the counts and percent were used and the Chi-square test was used for comparing the difference. We used the Kaplan–Meier to compare the survival curve of high RDW group and low RDW group. The univariate and multivariate Cox proportional hazard regression were used to estimate the relationship between RDW level and prognosis of patients with osteosarcoma, respectively. The hazard risk (HR) and 95% confidence interval (CI) were calculated. In the multivariate analysis, the following variables were adjusted: sex, age, body mass index, history of smoking, history of diseases, tumor size (≤5 vs >5 cm), clinical stage (I, II, III), the location of tumor (femur, tibia, humeral), metastasis or not, pathology types (osteoblastic, chondroblast, fibroblastic and telangiectatic) and response to chemotherapy (good vs poor) and parameters of blood routing examination. All analyses were performed using the SPSS 23.0 and GraphPad Prism 8.0. *P*<0.05 was considered statistically significant.

## Results

### Baseline data of study population

The present study finally included 271 patients with osteosarcoma with median follow-up time of 24.2 months (3–69 months). The mean age was 53.0 ± 9.29 years. There were 172 males (63.5%) and 99 females (36.5%). The smoking rate of these patients was 25.4% (*n*=69); 39.9% of all patients were at stage I/II and 163 patients at stage III. Other stage was absent. The location of tumor was distributed in femur (49.4%), tibia (32.1%) and humeral (18.5%). The types of pathology consisted of four types: osteoblastic, chondroblast, fibroblastic and telangiectasia. The ratios of four types were 38.7, 29.1, 25.5 and 6.6%, respectively. A total of 41.3% of patients had distant metastasis. Most patients have good response to chemotherapy (84.1%). The study population were divided into two groups according to the median of RDW (median = 13.6).

### Relationship between RDW and clinicopathological parameters

According to the RDW median, 135 patients belonged to the low RDW group, and 136 patients belonged to high RDW group. The Table 1 presented the comparisons of clinicopathological characteristics between low RDW and high RDW groups. There were significant differences in age (*P*=0.871), male ratio (*P*=0.180), body mass index (BMI) (*P*=0.053) and smoking ratio (*P*=0.778). Compared with low RDW group, the high RDW group tend to have metastasis (50 vs 32.6%, *P*=0.004). The high RDW have higher poor response rate to chemotherapy compared with the low RDW group (24.3 vs 7.4%, *P*=0.000). There was slightly significant difference in the types of pathology (χ^2^ = 8.059, *P*=0.045).

**Table 1:** Relationship between RDW and clinicopathological characteristics in patients with osteosarcoma

Parameters	≥Median (*n*=136)	<Median (*n*=135)	χ^2^/t	*P*
Age (years)	53.1 ± 9.3	52.9 ± 9.3	0.163	0.871
Male (%)	91 (67.4%)	81 (59.6%)	1.800	0.180
BMI (kg/m^2^)	21.7 ± 2.6	22.4 ± 27	1.943	0.053
Smoking (*n*, %)	36 (8.7%)	33 (8.0%)	0.079	0.778
Tumor size, cm			1.360	0.244
≤5	70 (51.5%)	79 (58.5%)		
>5	66 (48.5%)	56 (41.5%)		
Clinical stage			1.086	0.297
I/II	50 (36.8%)	58 (43.0%)		
III	86 (63.2%)	77 (57.0%)		
Location			4.297	0.117
Femur	68 (50.0%)	66 (48.9%)		
Tibia	49 (36.0%)	38 (28.1%)		
Humeral	19 (14.0%)	31 (23.0%)		
Metastasis			8.466	0.004
Absent	68 (50.0%)	91 (67.4%)		
Present	68 (50.0%)	44 (32.6%)		
Pathology			8.059	0.045
Osteoblastic	52 (38.2%)	53 (39.3%)		
Chondroblast	49 (36.0%)	30 (22.2%)		
Fibroblastic	27 (19.9%)	42 (31.1%)		
Telangiectatic	8 (5.9%)	10 (7.4%)		
Response to chemotherapy			14.422	0.000
Good	103 (75.7%)	125 (92.6%)		
Poor	33 (24.3%)	10 (7.4%)		

We also compared the clinical hematology parameters between two groups. Compared with low RDW group, the high RDW group tended to have higher high-sensitivity CRP (7.6 ± 4.9 vs 5.5 ± 4.5, t = 3.727, *P*=0.000). Of course, the RDW level was significantly higher than that in the low group (15.8 ± 7.8 vs 13.4 ± 7.1, t = 2.684, *P*=0.008). The counts of lymphocyte in the high RDW group was higher than that in the low RDW group (2.2 ± 0.5 vs 2.1 ± 0.4, t = 1.996, *P*=0.047). No significant differences were observed in red blood cell (*P*=0.182), white blood cell (*P*=0.450), hemoglobin (*P*=0.617), platelet (*P*=0.206), TG (*P*=0.508), LDL-C (*P*=0.309), HDL-C (*P*=0.428), total cholesterol (*P*=0.179). The details of all results are presented in the Table 2.

**Table 2:** Correlations between pre-treatment RDW and clinical hematology parameter in patients with osteosarcoma

Parameters	>Median	≤Median	*t*	*P*
Red blood cell, ×10^12^	6.2 ± 1.6	6.4 ± 1.7	1.339	0.182
White blood cell, ×10^9^	8.1 ± 3.4	8.5 ± 3.5	0.756	0.450
Hemoglobin, g/l	130.0 ± 14.1	129.2 ± 11.2	0.501	0.617
Platelet, ×10^9^/l	224.4 ± 63.0	234.1 ± 61.6	1.266	0.206
Lymphocyte, 10^9^/l	2.2 ± 0.5	2.1 ± 0.4	1.996	0.047
Total cholesterol, mmol/l	1.2 ± 0.9	1.3 ± 0.5	1.346	0.179
TG, mmol/l	1.4 ± 1.0	1.4 ± 0.9	0.663	0.508
HDL-C, mmol/l	1.4 ± 0.4	1.4 ± 0.34	4.020	0.428
LDL-C, mmol/l	3.1 ± 0.6	3.0 ± 0.6	0.794	0.309
High-sensitivity CRP, mg/l	7.6 ± 4.9	5.5 ± 4.5	3.727	0.000
RDW (%)	15.8 ± 7.8	13.4 ± 7.1	2.684	0.008

### RDW and prognosis of patients with osteosarcoma

**Figure 1:**
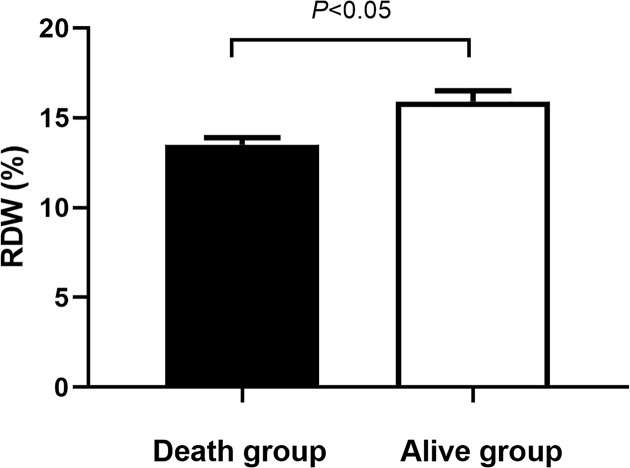
Comparison of RDW level between death group and alive group

**Figure 2:**
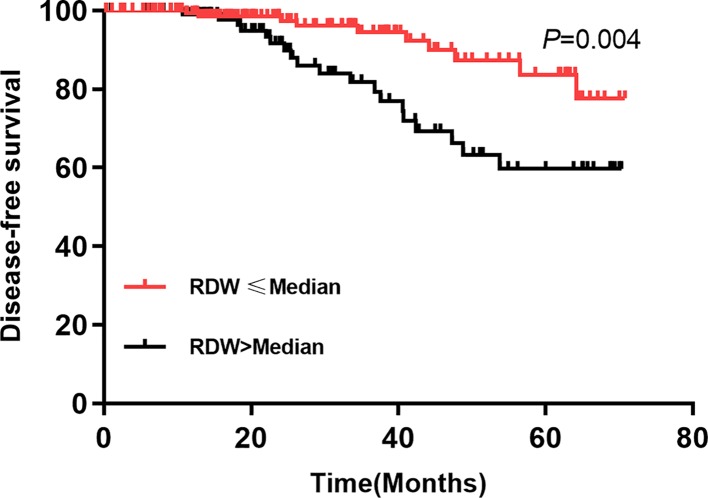
Comparison of OS between high RDW and low RDW groups in patients with osteosarcoma

We first compared the RDW level between alive group and death group. The significant difference was found between two groups (*P*<0.05, Figure 1). We further performed the Kaplan–Meier analysis (Figure 2). The survival curve of high RDW group was poorer than that in the low RDW group (*P*=0.020). The univariate and multivariate cox regression analyses were also conducted. The results were presented in the Tables 3 and 4, respectively. The univariate Cox analysis indicated that patients with RDW ≥ median had higher risk of poor prognosis compared with those who had RDW level < median (HR = 2.41, 95% CI: 1.51–3.83, *P*=0.000). Furthermore, patients with poor response to chemotherapy had poorer prognosis (HR = 2.36, 95% CI: 1.67–4.25, *P*=0.000). The advanced stage was positively correlated with poor prognosis (HR = 1.95, 95% CI: 1.20–3.816, *P*=0.007). Metastasis was also one of the factors affecting prognosis (HR = 2.15, 95% CI: 1.39–3.34, *P*=0.001). Other parameters were not related to prognosis of patients with osteosarcoma (*P*>0.05).

**Table 3:** Univariable cox regression analysis of OS for patients with osteosarcoma

Parameters	*B*	S.E.	Wald χ^2^	*P*	HR	95% CI
Age (years)	−0.017	0.012	2.145	0.143	0.98	0.96–1.01
BMI (kg/m^2^)	−0.020	0.044	0.206	0.650	0.98	0.90–1.07
Smoking (Yes vs No)	0.048	0.312	0.024	0.878	1.05	0.57–1.94
Tumor size (>5 vs ≤5 cm)	−0.271	0.225	1.455	0.228	0.76	0.49-1.19
Clinical Stage (III vs I/II)	0.667	0.246	7.349	0.007	1.95	1.20-3.16
Location, Femur (Reference)						
Tibia	−0.188	0.252	0.220	0.639	0.89	0.54-1.46
Humeral	0.320	0.296	1.170	0.279	1.38	0.77-2.46
Metastasis (Present vs absent)	0.765	0.225	11.622	0.001	2.15	1.39-3.34
Pathology, Osteoblastic (Reference)						
Chondroblast	−0.057	0.296	0.045	0.832	0.95	0.56-1.60
Fibroblastic	−0.117	0.306	0.147	0.702	0.89	0.49-1.62
Telangiectatic	0.525	0.383	1.88	0.170	1.69	0.80-3.58
Response to chemotherapy (Poor vs Good)	0.978	0.239	16.775	0.000	2.36	1.67-4.25
RDW (≥median vs <median)	0.879	0.237	13.768	0.000	2.41	1.51-3.83
Red blood cell, ×10^12^	−0.082	0.072	1.3060	0.253	0.92	0.80-1.06
White blood cell, ×10^9^	0.033	0.033	0.978	0.323	1.03	0.97-1.10
Hemoglobin, g/l	0.003	0.009	0.105	0.746	1.00	0.99-1.02
Platelet, ×10^9^/l	−0.003	0.002	3.534	0.060	0.99	0.99-1.00
Lymphocyte, 10^9^/l	0.001	0.243	0.000	0.998	1.00	0.62-1.61
Total cholesterol, mmol/l	0.080	0.119	0.447	0.504	1.08	0.86-1.37
TG, mmol/l	−0.186	0.115	2.607	0.106	0.83	0.66-1.04
HDL-C, mmol/l	0.020	0.183	0.012	0.912	1.02	0.71-1.46
LDL-C, mmol/l	−0.295	0.276	1.146	0.284	0.74	0.43-1.28
High-sensitivity CRP, mg/l	0.013	0.023	0.300	0.584	1.01	0.97-1.06

**Table 4:** Multivariable cox regression analysis of OS for patients with osteosarcoma

Parameters	*B*	S.E.	Waldχ^2^	*P*	HR	95%CI
Stage (III vs I/II)	0.588	0.250	5.524	0.019	1.80	1.10-2.94
Metastasis (Present vs Absent)	0.866	0.230	14.217	0.001	2.38	1.52-3.73
Response to chemotherapy (Poor vs Good)	0.993	0.249	15.966	0.000	2.70	1.66-4.39
RDW (≥median vs <median))	0.504	0.223	5.122	0.024	1.66	1.07-2.56

After adjusting some potential cofounding factors, the high RDW was still associated with poor prognosis (HR = 1.66, 95% CI: 1.07–2.56, *P*=0.024). The advanced stage, metastasis, poor response to chemotherapy were also independently related to poor prognosis of patients with osteosarcoma. The HRs and 95% CI were 1.80 (95% CI: 1.10–2.94, *P*=0.019), 2.38 (95% CI: 1.52–3.73, *P*=0.001) and 2.70 (95% CI: 1.66–4.39, *P*=0.000), respectively.

## Discussion

Our results indicated that elevated RDW level was positively associated with metastasis and poor response to chemotherapy. It is known that inflammation status in the tumor microenvironment was involved in tumor growth, invasion and metastasis [16]. Previous studies have suggested that some inflammatory factors such as CRP, interleukin-6 and neutrophil to lymphocyte ratio can predict the prognosis in cancer patients [17–20]. In this study, we also found the high RDW level tend to have higher CRP and elevated lymphocyte. The multivariate analysis further suggested that the increased RDW level was independently related to poor prognosis in patients with osteosarcoma. The present results indicated that we should give some attention to these patients who have elevated RDW level.

In recent years, more and more studies have found the relationship between RDW and prognosis of tumor patients. In a retrospective case–control study, Spell et al. [21] assessed the results of 494 patients who received sigmoidoscopy with negative results for 5 years follow-up, and found that 46% of the patients (225 cases) were eventually diagnosed with colorectal cancer. Patients with colorectal cancer had a higher mean RDW than the control group, and the mean RDW was found to be 16.8% in the right lesion patients, 15.0% in the left lesion patients and 13.3% in the normal control group (reference upper limit of erythrocyte distribution width: 14.1%). Accordingly, the sensitivity and specificity of RDW in the detection of colorectal cancer were 0.69 and 0.88, respectively. Ozkalemkas et al. [22] assessed the pathological diagnosis of bone marrow in nine cases (according to the pathological tumor location: five cases of stomach, three cases of gastrointestinal tract, three cases of prostate cancer, two cases of lung, one case of muscle and five cases of unknown), and found red blood cell distribution width in patients were higher than local limit detection reference value in all cases. By including 253 patients admitted to hospital due to unexplained weight loss, Baicus et al. [23] found that 61 patients (accounting for 24% of all patients) were eventually diagnosed as malignant tumors. The RDW of these patients who were later diagnosed with tumor was higher than that of non-tumor patients (mean value: 15.1 vs 14.6%, *P*=0.022), and the other tumor-related indicators were erythrocyte sedimentation rate, CRP, hemoglobin level and serum iron equality. In the multivariate analysis, only red cell sedimentation rate was associated with tumor [23]. Koma et al. [24], reviewed data from 332 lung cancer patients, found that high cell distribution width (>15%) and have higher stage, worse physical status score, higher CRP and WBC count, lower blood albumin levels and higher levels of cytokeratin, and suggested red blood cell distribution width can be used as a prognosis marker of tumor patients. Our results provide further evidence support for the role of RDW in the cancers.

Although the potential mechanism remains unclear, inflammation may partly explain this relationship. Previous studies have shown that inflammation plays an important role in promoting tumor development and metastasis [25]. Inflammation produces a large number of inflammatory mediators, which can not only directly act on normal cells to generate mutations and cause tumor occurrence, but also promote tumor proliferation, induce angiogenesis and tissue remodeling, and generate immune tolerance by regulating local microenvironment of tumor, so as to jointly promote the occurrence, development and metastasis of tumor. At the same time, tumor cells can also release inflammatory mediators to maintain the state of inflammatory response for their own development [26]. The inflammation also caused changes in red blood cell maturation by disturbing the red cell membrane, inducing increased RDW. Our results indicated the RDW was associated the inflammatory biomarker, CRP. This results further suggested that RDW plays an important role in the inflammatory process [27]. The oxidative stress may also be involved in this process. As we all know, oxygen is required for mitochondria to produce adenosine triphosphate by oxidative phosphorylation. At the same time, due to the chemical activity of oxygen molecules, reactive oxygen species (ROS) are easily formed. ROS is a normal product in cell metabolism, including superoxide, peroxides and hydroxyl radicals [28]. The electron transport chain in mitochondrial metabolism is the main source of ROS. ROS plays an important role in the development of tumors. *In vitro* studies have shown that ROS production in tumor cells is much faster than normal cells. A certain level of ROS increases mutation frequency activates proliferation-related signaling pathways, and promotes tumor progression [29,30]. Previous study reported that elevated RDW level is associated with oxidative stress and inflammation in a canine model of rapid atrial pacing [31]. Just like inflammation, the status of oxidative stress may change red cell survival and induced elevated RDW level.

There are several study limitations. First, as a retrospective study, we did not explore the relationship between RDW and disease-free survival because the accurate time point can be confirmed based on present follow-up methods. Second, tumors are caused by different kinds of gene and environment factors. We extracted the most relevant factors in this analysis. Some factors associated with osteosarcoma were not included, which may have some influences on estimated results. We have excluded some important factors that affected RDW level according to the criteria for inclusion. Third, this is a single-centered retrospective design that may have some bias in data collection and sample selection. Finally, we did not explore the molecular mechanism. Study with multicenter design and larger sample size is needed.

In conclusion, as a routinely available marker of the systemic inflammatory response, the pretreatment RDW elevation was associated with poor OS in patients with osteosarcoma and can be an independent predictor of poor prognosis. As a cost-effective and easily calculated index almost universally available using this common hematological parameter, RDW can improve risk evaluation. Further research is required for the specific mechanism.

## Abbreviations

CI: confidence interval
CRP: C-reactive protein
HDL-C: high-density lipoprotein cholesterol
HR: hazard risk
LDL-C: low-density lipoprotein cholesterol
OS: overall survival
RDW: red cell distribution width
ROS: reactive oxygen species
TC: total cholesterol
TG: triglyceride
